# OA23 Celebrating the achievements and efforts of children, young people and their siblings – a charity award badge scheme for families living with paediatric rheumatological conditions in the UK

**DOI:** 10.1093/rap/rkac066.023

**Published:** 2022-09-28

**Authors:** Emily Earle

**Affiliations:** CCAA - Kids With Arthritis, London, United Kingdom

## Abstract

**Introduction/Background:**

Many children and young people with Juvenile Idiopathic Arthritis (JIA) and other rheumatological conditions can face extra challenges as they grow up. These include coping with their condition, treatments and treatment side effects but also attending school and achieving in their chosen hobbies. Families often play a vital role in supporting children at home and so these challenges are rarely seen by others. The aim was to create a way to support families in recognising, encouraging and celebrating the efforts of their children and young people, as well as siblings, in relation to managing life with their condition.

**Description/Method:**

The Children’s Chronic Arthritis Association (CCAA) is a charity offering support to families living with JIA in the UK. CCAA decided to prioritise the scheme during the COVID-19 pandemic to offer another avenue of support direct into families’ homes. During this time, some families had less contact with their care teams, many children were feeling isolated while not at school and we were not able to offer our usual peer support activities. The concept was put, via a survey, to the group of 38 CCAA local area parent representatives who represent families from across the UK. Incorporating their feedback, we developed our initial offering of six badges – Effort, Courage, Raising Awareness, Fundraising, Supportive Sibling and Active Achievement. We aimed to promote CCAA core values. We wanted to focus less on success or achieving goals but instead to celebrate trying and persevering. We developed a website page to promote the badges including a nomination form, rules, a guide to help families find the award badge best suited to their child’s needs and a set of Frequently Asked Questions (FAQs). Designs were chosen to appeal visually and to be collectible. We added a lanyard on which to collect and keep the badges and a certificate delivered in environmentally friendly packaging.  The scheme was launched in April 2021. We established a weekly ‘Monday Badge Story’ social media post where photos and stories of badge recipients were shared (with consent from families).  Early in 2022 we added one further award for Research as we felt this was lacking from the original offering and was another area that we wanted to encourage our families with. On the first anniversary of the scheme in April 2022 we added a medal for the end of the lanyard for any child who had collected eight awards.

**Discussion/Results:**

Selected statistics from the first year of the scheme being live:

Some aspects of the scheme being live during the first year have provided learning for us in the areas of support and raising awareness. In terms of support, the scheme offered support more widely than just to the child or young person alone. Many nominators gave lengthy reasons for nominating the child or young person suggesting that the nomination process itself is cathartic for parents and families and can help them feel that they are being heard. Children, young people and their families also appreciate reading the badge stories of others like them and it may help them feel less alone. We have also been able to support the work of Healthcare Professionals who have appreciated the opportunity to reward and incentivise their patients. With regards to raising awareness, the accessibility of the nomination process to all has generated a wide range of nominators (as detailed in the table above) which has naturally aided raising awareness of JIA across the communities we support. Our ‘Badge Story’ social media posts have been a good way to help increase awareness of paediatric rheumatological conditions in children and young people more widely still.

**Key learning points/Conclusion:**

Positive feedback sent from families and clinicians shows us that the scheme has been well received. As one Clinical Nurse Specialist said, “I have recently come into post and have used your award badge scheme a few times now, the kids love it!” Comments from parents show the collectible side to the scheme: “They are looking forward to finding out how to get more badges.” They also reflect how the badges can help increase positivity and self-esteem: “The badges are a fabulous idea to lift their moods,” and “She is thrilled and felt so proud.”  They also show how they can promote and incentivise positive behaviour change and coping skills: “Her badges last week really helped with her injection on Friday.” Developing this scheme has allowed us to offer support directly to families in an individual way that is tailored to the needs of each family. The support is driven by the family themselves or their medical team or others who know them well. Families have particularly appreciated the ability to reward siblings whose needs are often overlooked.  The scheme ensures that families can self-access an entirely different type of support to the face-to-face residential weekends and local meet ups or the online support groups we already offer. This new line of support has the added advantage of being available and easily accessible whenever it is needed. An unanticipated benefit is that we have also been able to increase our reach to some families who have not engaged with the charity previously by accepting nominations from healthcare professionals who have contact with these families. Going forward, at the end of the second year of being live, we aim to more formally evaluate the scheme and measure its impact.

**OA23 Table 1:** 

First Year of the CCAA Award Badge Scheme	
Badges Awarded	394
Nominators were:	67% Mums 13% Clinical Nurse Specialists 4% Grandparents 2% Dads 2% Teachers (Others included family friends, neighbours, siblings, Aunties, Physios, Best friends, School Librarians, Consultant Paediatric Rheumatologists, Nurses, Sports/Activity Instructors, Youth Workers, Research Coordinators)
Most popular badge	Courage 51%
Age range of award nominees	1 – 23 years

